# Effects of Autonomy‐Supportive Music Instruction on Student Wellness: A Cluster‐Randomized Test of Psychological Need Satisfaction and Autonomous Motivation as Motivational Mechanisms

**DOI:** 10.1002/pchj.70127

**Published:** 2026-11-10

**Authors:** Jing Chen

**Affiliations:** ^1^ College of Arts and Design Zhengjiang A&F University Hangzhou Zhengjiang China

**Keywords:** autonomous motivation, autonomy‐supportive instruction, experimental design, music education, psychological need satisfaction, student wellness

## Abstract

This study examines the effects of autonomy‐supportive music instruction on student wellness and investigates the underlying motivational mechanisms through which such instruction may promote wellness in university music education. Drawing on self‐determination theory, a cluster‐randomized experimental design was implemented among undergraduate music students in China. Intact classes were randomly assigned to an autonomy‐supportive instructional condition or a control condition. Data were collected at three time points using paper‐based questionnaires, and multilevel analyses were used to account for the nested structure of students within classes. The results indicate that autonomy‐supportive music instruction increased students' psychological need satisfaction and autonomous motivation. In turn, both psychological need satisfaction and autonomous motivation were positively associated with post‐intervention student wellness. Multilevel mediation analyses using bootstrapping revealed significant indirect effects of autonomy‐supportive instruction on student wellness through both psychological need satisfaction and autonomous motivation, whereas the direct effect was no longer significant after the mediators were included. Overall, the findings provide experimental evidence that autonomy‐supportive music instruction can promote student wellness by shaping students' motivational experiences. The study extends self‐determination theory to a performance‐oriented, non‐Western music education context and highlights psychological need satisfaction and autonomous motivation as distinct motivational pathways linking instructional practice to student wellness.

## 1 Introduction

Across higher education systems, concerns about student wellness have intensified as academic demands, performance pressures, and psychological strain increasingly intersect with learning experiences. Recent evidence indicates that university students worldwide report rising levels of stress, anxiety, and emotional exhaustion, prompting growing calls for educational environments that support not only academic development but also psychological well‐being (Fülöp et al. 2025; Klimova and Pikhart 2025; Pérez‐Jorge et al. 2025; Solms et al. 2025). In this regard, instructional practices are increasingly recognized as a central contextual factor shaping students' day‐to‐day psychological experiences, particularly in disciplines characterized by high evaluative intensity and sustained performance expectations. Music education represents one such context, where public performance, continuous feedback, and identity‐relevant evaluation are integral to learning yet may simultaneously heighten vulnerability to stress and diminished well‐being (Futch et al. 2025; McNickle 2022). Music students may also encounter sustained practice demands, performance anxiety, perfectionistic expectations, and repeated judgments of their competence, making the instructional environment particularly consequential for their psychological functioning (Arbinaga 2023; Bonneville‐Roussy et al. 2020; Hatfield et al. 2025; Koehler et al. 2023). Accordingly, student wellness in music education cannot be considered separately from the pedagogical conditions under which students practice, perform, receive feedback, and develop their musical identities (Bonneville‐Roussy and Evans 2025; Butler 2022).

From a theoretical standpoint, self‐determination theory (SDT) provides a robust framework for explaining how instructional environments influence student wellness through motivational processes. According to Deci and Ryan (2000), social contexts that support individuals' basic psychological needs for autonomy, competence, and relatedness foster more internalized forms of motivation and higher levels of psychological well‐being. Building on this perspective, autonomy‐supportive teaching has been conceptualized as an instructional approach that emphasizes meaningful choice, acknowledgment of learners' perspectives, and the provision of rationales, rather than reliance on control or external pressure (Xu and Li 2025). In music instruction, such support may involve allowing students meaningful input into repertoire or learning strategies, acknowledging their artistic perspectives, providing informative rather than pressuring feedback, and explaining the rationale for demanding technical activities (Bonneville‐Roussy et al. 2020; Butler 2022; Hendriks et al. 2023). Accumulating evidence across educational contexts indicates that autonomy‐supportive instruction is associated with enhanced engagement, persistence, and adaptive learning outcomes, suggesting that instructional style constitutes a powerful motivational resource embedded within everyday classroom interactions (Bonneville‐Roussy and Evans 2025; Butler 2022; Hendriks et al. 2023). Intervention research similarly indicates that even relatively brief autonomy‐supportive instructional programs can improve students' psychological need satisfaction and autonomous motivation, although such evidence has emerged predominantly outside university music education (Huang et al. 2025; Matos et al. 2025; Tilga et al. 2021).

Despite this growing consensus, empirical research has largely prioritized academic achievement, engagement, or performance outcomes, with student wellness receiving comparatively limited attention. Although some studies document positive associations between autonomy‐supportive teaching and well‐being indicators, these findings are predominantly derived from cross‐sectional designs, limiting causal inference and obscuring the psychological processes through which instructional practices may affect wellness (Bond 2024; Utomo 2025; Xu and Li 2025). Within music education, existing research has also frequently relied on observational, correlational, or non‐randomized evidence, leaving uncertainty about whether autonomy‐supportive instructional practices themselves produce changes in students' wellness (Bonneville‐Roussy et al. 2020; Bond 2024; Xu and Li 2025). Moreover, wellness is often treated as a distal or ancillary outcome rather than as a central educational objective, particularly within performance‐oriented domains such as music education. This emphasis is consequential because technically successful learning may coexist with anxiety, emotional exhaustion, or diminished psychological functioning when students experience sustained evaluative pressure (Arbinaga 2023; Bonneville‐Roussy and Evans 2025; Hatfield et al. 2025). As a result, there remains a critical gap in understanding whether autonomy‐supportive instruction can causally enhance student wellness and through which motivational mechanisms such effects are realized. More specifically, experimental, longitudinal, and multilevel studies remain limited in this literature, even though music students are commonly nested within classes and share the instructional practices of the same teacher (Li et al. 2024; Rutterford et al. 2015).

Extending prior work, psychological need satisfaction represents a theoretically grounded mechanism linking instructional practices to student wellness. Need satisfaction reflects the extent to which learners experience volition, effectiveness, and social connection within learning environments (Hatfield et al. 2025), and has been consistently linked to indicators of vitality, life satisfaction, and mental health across educational and occupational contexts (Harrison et al. 2025; Martela et al. 2023; Schutte and Malouff 2021). In music education specifically, where students' sense of competence and autonomy is frequently challenged by evaluative norms and comparison, need satisfaction may play a particularly salient role in sustaining psychological well‐being. Autonomy may be constrained when instructors prescribe how students should interpret, practice, or perform music; competence may be challenged by repeated evaluation and comparison; and relatedness may depend on the quality of interactions with instructors and peers (Bonneville‐Roussy et al. 2020; Hatfield et al. 2025; Muth and Lüftenegger 2024). Yet, empirical research examining need satisfaction as a mediating mechanism between instructional style and wellness remains limited, especially within experimental designs. Although autonomy, competence, and relatedness are conceptually distinguishable, SDT also treats their collective satisfaction as a general indicator of the extent to which a social environment supports optimal psychological functioning (Deci and Ryan 2000; Martela et al. 2023; Schutte and Malouff 2021). Accordingly, the present study examines overall psychological need satisfaction because its primary purpose is to test the general need‐based process through which autonomy‐supportive music instruction may contribute to student wellness.

Building on this logic, autonomous motivation offers an additional explanatory pathway through which instructional practices may influence student wellness. Autonomous motivation reflects engagement driven by intrinsic interest or personal value, rather than external demands or pressure (Bonneville‐Roussy and Evans 2025), and has been shown to predict lower burnout, reduced stress, and greater psychological resilience among students (Finnerty et al. 2022; Koehler et al. 2023; Li 2024). This motivational distinction is particularly relevant in music education because sustained practice may be undertaken either for enjoyment and personally endorsed musical goals or in response to evaluation, obligation, and external expectations (Bonneville‐Roussy and Evans 2025; Koehler et al. 2023). Instructional contexts that support autonomy are more likely to facilitate such self‐determined motivation, thereby promoting not only sustained engagement but also positive psychological functioning. However, few studies have simultaneously examined psychological need satisfaction and autonomous motivation as sequential or parallel mechanisms linking autonomy‐supportive instruction to student wellness, particularly using rigorous experimental methods. Although SDT suggests that need satisfaction can facilitate the internalization of motivation, need satisfaction and autonomous motivation remain theoretically distinct experiences: the former concerns whether students' psychological needs are fulfilled, whereas the latter concerns why students engage in learning activities (Deci and Ryan 2000; Ryan and Deci 2017). The present study therefore examines them as parallel mediating mechanisms to determine whether autonomy‐supportive music instruction is linked to wellness through students' need experiences, their self‐endorsed reasons for learning, or both.

From a contextual perspective, the majority of autonomy‐supportive teaching research has been conducted in Western educational settings (Hinnersmann et al. 2020), raising questions about the generalizability of these findings to non‐Western contexts. Although SDT posits that basic psychological needs are universal, cultural norms surrounding authority, hierarchy, and instruction may shape how autonomy‐supportive practices are enacted and experienced (Bonneville‐Roussy et al. 2020; Bonneville‐Roussy and Evans 2025). Empirical evidence from East Asian higher education contexts remains comparatively sparse, particularly in disciplines such as music education, where traditional instructional norms may emphasize control and conformity (Lam 2023). In such settings, autonomy support should not be equated with the absence of structure or unrestricted student independence; rather, it concerns enabling students to experience volition and personal endorsement within clearly structured instructional activities (Reeve and Cheon 2021; Patzak and Zhang 2025). Addressing this gap is essential for advancing cross‐cultural motivation research and for testing the boundary conditions of autonomy‐supportive pedagogy. Evidence that autonomy‐supportive instruction benefits Chinese music students would therefore support the contextual applicability of SDT while also showing how its principles operate within a comparatively hierarchical and performance‐oriented educational environment (Lam 2023; Xu and Li 2025).

## 2 The Present Study

Against this backdrop, the present study seeks to examine the effects of autonomy‐supportive music instruction on student wellness and to elucidate the motivational mechanisms underlying this relationship. Using a cluster‐randomized experimental design with university music students in China, the study tests psychological need satisfaction and autonomous motivation as mediators linking instructional style to student wellness. The study focuses specifically on university music classes because students within these settings share instructional conditions while facing sustained practice, evaluation, and performance demands that may influence both their motivation and psychological functioning (Bonneville‐Roussy and Evans 2025; Hatfield et al. 2025). Intact classes are therefore randomized to minimize contamination between instructional conditions and to preserve the naturally nested structure of music instruction (Li et al. 2024; Rutterford et al. 2015).

By integrating experimental manipulation, multilevel modeling, and longitudinal measurement, the study aims to provide evidence for how autonomy‐supportive teaching practices contribute to student well‐being in performance‐oriented higher education contexts. Its central theoretical contribution lies in testing whether an experimentally created autonomy‐supportive music environment promotes student wellness through two distinct SDT‐based mechanisms: the satisfaction of students' basic psychological needs and the development of autonomous reasons for engaging in music learning. In doing so, the study moves beyond merely transferring SDT to another setting by clarifying how instructional practice is connected to wellness within a non‐Western, performance‐intensive music education context.

## 3 Hypotheses Development

### 3.1 Autonomy‐Supportive Instruction and Student Wellness

Within contemporary higher education research, student wellness is increasingly understood as an outcome shaped by instructional environments rather than as an individual disposition alone. In this milieu, Xu and Li (2025) showed that students' psychological well‐being is systematically linked to how academic demands are structured and communicated, particularly in contexts characterized by continuous evaluation. Building on this evidence, Butler (2022) demonstrated that pedagogical environments emphasizing support and agency are associated with lower psychological distress and higher subjective well‐being among university students. However, much of this evidence is correlational, making it difficult to determine whether autonomy‐supportive instructional practices themselves produce improvements in wellness or merely coexist with more favorable student experiences (Bond 2024; Xu and Li 2025).

From a motivational perspective, instructional environments that rely heavily on control, pressure, and compliance have been shown to undermine students' psychological functioning. For instance, Hendriks et al. (2023) reported that controlling instructional behaviors predicted emotional exhaustion and maladaptive engagement patterns, even after accounting for workload. In contrast, Reeve and Cheon (2021) argued that autonomy‐supportive instructional climates reduce evaluative threat and foster adaptive psychological outcomes by regulating how demands are experienced by learners. From the perspective of music education, Bonneville‐Roussy et al. (2020) documented elevated levels of anxiety and stress among music students, attributing these outcomes to persistent performance evaluation and identity‐relevant competence judgments. Similarly, Bonneville‐Roussy and Evans (2025) found that music students reported higher psychological strain than peers in non‐performance disciplines, highlighting the mental health risks embedded within performance‐oriented learning environments. These pressures are especially consequential in music education because students are often evaluated publicly, compare their competence with peers, and receive feedback that may be interpreted as a judgment of both performance and artistic identity (Arbinaga 2023; Hatfield et al. 2025). Under such conditions, instructional practices that mitigate pressure and support students' agency may play a critical role in sustaining wellness.

Against this backdrop, autonomy‐supportive instruction has been proposed as a pedagogical approach capable of buffering psychological strain by fostering supportive learning climates. Qin and Fitri (2024) posited that instructional environments acknowledging learners' perspectives and providing meaningful rationales promote psychological functioning by reducing external pressure. Empirical work across educational contexts supports this claim, showing that autonomy‐supportive teaching is associated with higher vitality and emotional well‐being (Pérez‐Jorge et al. 2025; Solms et al. 2025). Intervention‐based evidence further suggests that autonomy‐supportive practices can improve students' motivational and psychological experiences, although such studies have largely been conducted outside university music education (Cheon et al. 2023; Matos et al. 2025; Tilga et al. 2021). This distinction is important because the evaluative, performance‐intensive nature of music learning may shape how students experience instructional autonomy support and its consequences for wellness (Bonneville‐Roussy and Evans 2025; Xu and Li 2025). Hence, it is hypothesized that:

Hypothesis 1 *Autonomy‐supportive music instruction has a positive effect on student wellness*.

### 3.2 Autonomy‐Supportive Instruction and Psychological Need Satisfaction

Within SDT, psychological need satisfaction is conceptualized as the foundational mechanism linking social contexts to psychological functioning. Deci and Ryan (2000) posited that satisfaction of autonomy, competence, and relatedness constitutes a basic requirement for mental health, such that environments supporting these needs facilitate well‐being, whereas need‐thwarting environments undermine it. This theoretical proposition has been subjected to extensive empirical scrutiny across domains. Hood and Patton (2022), examining organizational contexts, demonstrated that employees' satisfaction of psychological needs predicted vitality and psychological adjustment beyond job characteristics. Extending this logic to educational settings, Fülöp et al. (2025) and Klimova and Pikhart (2025) found that students who experienced greater need satisfaction reported higher well‐being and emotional engagement, even in academically demanding environments. Hudays et al. (2024) further showed that need satisfaction consistently predicts mental health outcomes across work and education, reinforcing its role as a generalizable psychological mechanism. More broadly, evidence across countries indicates that satisfaction of autonomy, competence, and relatedness is closely associated with well‐being, meaning, and lower depressive symptoms, supporting the relevance of need satisfaction across diverse social and cultural settings (Martela et al. 2023; Schutte and Malouff 2021).

Moreover, instructional practices have been identified as a primary contextual source of need satisfaction. According to Butler (2022), autonomy‐supportive teaching fosters need satisfaction by enabling volitional engagement and affirming learners' competence. Similarly, Cheon et al. (2022) reported that autonomy‐supportive instructional behaviors predicted students' need satisfaction in both Western and East Asian samples, supporting the cross‐cultural relevance of this mechanism. More recently, Matos et al. (2025) showed that classrooms characterized by autonomy support exhibited systematically higher levels of student need satisfaction. This relationship is theoretically plausible because autonomy‐supportive instructors do more than provide choice: they acknowledge students' perspectives, offer meaningful rationales, communicate confidence in students' capabilities, and cultivate interpersonal involvement, thereby supporting autonomy, competence, and relatedness simultaneously (Deci and Ryan 2000; Reeve and Cheon 2021).

Intervention‐based evidence further strengthens this argument. For instance, Matos et al. (2025) demonstrated that training teachers to adopt autonomy‐supportive practices led to measurable increases in students' psychological need satisfaction. Similarly, Cheon et al. (2023) replicated these effects in experimental classroom interventions, providing evidence that instructional style directly shapes students' need experiences. Tilga et al. (2021) likewise showed that a brief autonomy‐supportive intervention improved students' basic psychological needs and motivational outcomes, suggesting that even relatively focused changes in instructional behavior can alter how learners experience their educational environment. However, such intervention evidence has emerged mainly from general education and physical activity settings, leaving the effects of autonomy‐supportive instruction on need satisfaction in university music education comparatively underexamined (Cheon et al. 2023; Tilga et al. 2021).

Within music education, evaluative norms and performance comparison frequently challenge students' perceptions of autonomy and competence. Muth and Lüftenegger (2024) argued that these conditions heighten vulnerability to need frustration. Music students may experience reduced autonomy when repertoire, interpretation, or practice routines are imposed; diminished competence when feedback is primarily comparative or judgmental; and weakened relatedness when instructional relationships are experienced as controlling or emotionally distant (Bonneville‐Roussy et al. 2020; Hatfield et al. 2025). Conversely, autonomy‐supportive music instruction can provide meaningful choice, informational feedback, and acknowledgment of students' artistic perspectives, thereby creating conditions more likely to satisfy all three psychological needs (Bonneville‐Roussy et al. 2020; Butler 2022; Xu and Li 2025). These findings suggest that autonomy‐supportive instruction may be particularly effective in enhancing need satisfaction in music learning contexts.

Hypothesis 2 *Autonomy‐supportive music instruction has a positive effect on students' psychological need satisfaction*.

### 3.3 Autonomy‐Supportive Instruction and Autonomous Motivation

Beyond shaping need satisfaction, instructional environments influence how students regulate their engagement. Autonomous motivation reflects engagement driven by intrinsic interest or personal value, rather than external pressure or obligation. Ryan and Deci (2017) argued that autonomous motivation represents a qualitatively superior form of regulation, associated with adaptive psychological outcomes and sustained engagement. Within SDT, autonomy‐supportive environments facilitate this form of motivation by helping students internalize the value of learning activities and experience their participation as self‐endorsed rather than externally imposed (Ryan and Deci 2017; Reeve and Cheon 2021).

Empirical research across higher education supports this distinction. For example, Wang et al. (2025) demonstrated that autonomous motivation predicts lower burnout and emotional exhaustion, whereas controlled motivation predicts maladaptive outcomes. Further, Patzak and Zhang (2025), in a large‐scale meta‐analysis, showed that autonomous motivation is consistently associated with psychological well‐being across educational settings. In addition, Howard, Slemp, and Wang (2025) reported that autonomy‐supportive teaching reliably predicts increases in autonomous motivation by facilitating internalization. Yang et al. (2022) provided further evidence that instructional autonomy support enhances students' autonomous regulation while reducing controlled forms of motivation. Intervention‐based findings also indicate that changes in instructional style can produce corresponding changes in students' autonomous motivation. Krpanec et al. (2024) demonstrated that autonomy‐supportive instructional interventions produced sustained increases in autonomous motivation, while Reeve and Cheon (2025) observed similar effects in classroom‐based settings. Likewise, Tilga et al. (2021) found that a brief autonomy‐supportive intervention improved students' motivational functioning, reinforcing the proposition that autonomous motivation can be shaped through deliberate changes in instructional practice.

In music education, autonomous motivation has been linked to sustained practice, adaptive performance experiences, and lower performance anxiety. Xu and Li (2025) found that music students with higher autonomous motivation exhibited more adaptive emotional responses to performance demands. Bonneville‐Roussy et al. (2020) similarly reported that autonomous regulation was associated with lower anxiety and greater psychological resilience among music students. This relationship is especially relevant because music learning typically requires prolonged and effortful practice; when students engage for enjoyment or personally valued goals, these demands may be experienced differently from practice driven primarily by evaluation, obligation, or external pressure (Bonneville‐Roussy and Evans 2025; Koehler et al. 2023). Autonomy‐supportive music instruction may therefore strengthen autonomous motivation by providing meaningful choice, acknowledging students' artistic preferences, and explaining the value of demanding learning activities (Bonneville‐Roussy et al. 2020; Butler 2022). These findings suggest that autonomy‐supportive music instruction is likely to enhance autonomous motivation.

Hypothesis 3 *Autonomy‐supportive music instruction has a positive effect on students' autonomous motivation*.

### 3.4 Psychological Need Satisfaction as a Mediating Mechanism

SDT explicitly posits psychological need satisfaction as a mediating process through which social contexts influence well‐being. Deci and Ryan (2000) argued that contextual effects on psychological outcomes operate indirectly via need satisfaction rather than directly. According to SDT, autonomy‐supportive environments facilitate well‐being because they create conditions in which individuals experience greater autonomy, competence, and relatedness, thereby promoting optimal psychological functioning rather than merely reducing external pressures (Deci and Ryan 2000; Ryan and Deci 2017). Empirical evidence across domains supports this mediational logic.

Parfyonova et al. (2019) demonstrated that need satisfaction mediated the relationship between managerial support and employee well‐being. In educational settings, Reeve and Cheon (2024) found that autonomy‐supportive teaching predicted well‐being indirectly through need satisfaction, with direct effects diminishing once need satisfaction was included. Howard, Slemp, and Wang (2025) reported similar patterns, showing that need satisfaction accounted for the association between instructional autonomy support and emotional engagement. Collectively, these findings suggest that the beneficial effects of autonomy‐supportive environments are not expected to emerge automatically; rather, they are transmitted through students' perceptions that their fundamental psychological needs are being fulfilled (Reeve and Cheon 2024).

Yoo et al. (2025) further argued that mediation through need satisfaction represents a robust explanatory pathway across contexts, rather than a context‐specific phenomenon. In music education, Matos et al. (2025) suggested that psychological strain experienced by music students reflects chronic need frustration arising from evaluative instructional climates. Similarly, Hatfield et al. (2025) reported that the satisfaction and frustration of basic psychological needs were closely associated with life satisfaction, somatic burden, and intentions to disengage among music students, highlighting the central role of need‐based experiences in this educational context. From an empirical perspective, Sharpe et al. (2024) linked performance pressure to reduced psychological functioning, suggesting that need satisfaction may explain how instructional practices influence wellness in this domain. Taken together, these findings indicate that autonomy‐supportive music instruction may improve student wellness not simply because it changes teaching behavior but because it transforms how students experience autonomy, competence, and relatedness within demanding music learning environments (Bonneville‐Roussy et al. 2020; Hatfield et al. 2025; Xu and Li 2025).

Despite these theoretical and empirical indications, mediation tests explicitly examining psychological need satisfaction in music education remain rare, particularly within experimental designs. Moreover, evidence based on cluster‐randomized, longitudinal, and multilevel research remains especially limited, despite the nested nature of music classrooms and the shared instructional experiences of students within the same class (Li et al. 2024; Rutterford et al. 2015). Consistent with SDT and prior mediation evidence, the present study proposes the following hypothesis:

Hypothesis 4 *Psychological need satisfaction mediates the relationship between autonomy‐supportive music instruction and student wellness*.

### 3.5 Autonomous Motivation as a Mediating Mechanism

Autonomous motivation represents a complementary pathway through which instructional environments may influence student wellness. Vega‐Díaz et al. (2025) demonstrated that motivational regulation mediates the relationship between contextual support and psychological outcomes, including stress and burnout. Lewczuk et al. (2022) similarly reported that autonomous motivation functions as a psychological resource buffering against emotional exhaustion. This mediating role reflects the proposition that supportive environments contribute to psychological functioning by enabling individuals to engage in activities for intrinsically satisfying or personally endorsed reasons rather than because of external pressure (Ryan and Deci 2017).

Instructional research provides further support for this mediational role. Hoff et al. (2024) showed that autonomy‐supportive teaching predicted students' psychological adjustment indirectly via autonomous motivation. Ryan and Deci (2017) argued that autonomy‐supportive contexts influence well‐being primarily by facilitating self‐endorsed engagement rather than by exerting direct effects. Accordingly, autonomy‐supportive instruction may enhance wellness not simply by making the learning environment more favorable, but by changing the reasons for which students participate and persist in demanding learning activities (Howard, Slemp, and Wang 2025; Yang et al. 2022). When students perceive their engagement as volitional and personally meaningful, the effort required by learning may be experienced as less pressuring and more psychologically sustainable (Patzak and Zhang 2025; Ryan and Deci 2017).

In music education, empirical studies suggest that motivational regulation is closely linked to emotional experiences during learning and performance. Cai and Lombaerts (2024) found that autonomous motivation mediated the relationship between learning environments and performance‐related affect. Related evidence indicates that autonomous motivation is associated with more adaptive emotional experiences, sustained practice, and greater well‐being among music learners and musicians (Bonneville‐Roussy and Evans 2025; Koehler et al. 2023; Xu and Li 2025). Similarly, Shang and Ma (2024) indicated that self‐determined motivation was associated with reduced anxiety and greater emotional stability among students. This pathway is particularly relevant in music education because intensive practice and repeated performance evaluation may be experienced either as personally valued engagement or as externally imposed obligation, with different implications for students' psychological functioning (Bonneville‐Roussy et al. 2020; Bonneville‐Roussy and Evans 2025). These findings imply that autonomous motivation may explain how autonomy‐supportive music instruction influences student wellness.

However, experimental studies explicitly testing this mediational pathway in music education are limited. Existing studies have largely examined motivational regulation through correlational or non‐randomized designs, leaving limited evidence about whether experimentally introduced autonomy‐supportive music instruction promotes wellness through changes in autonomous motivation (Bond 2024; Xu and Li 2025). Moreover, few studies have tested this indirect pathway while accounting for the nesting of students within shared instructional environments (Li et al. 2024; Rutterford et al. 2015). Addressing this gap, the present study advances the following hypothesis:

Hypothesis 5 *Autonomous motivation mediates the relationship between autonomy‐supportive music instruction and student wellness*.

Figure 1 shows the proposed conceptual model below.

**FIGURE 1:**
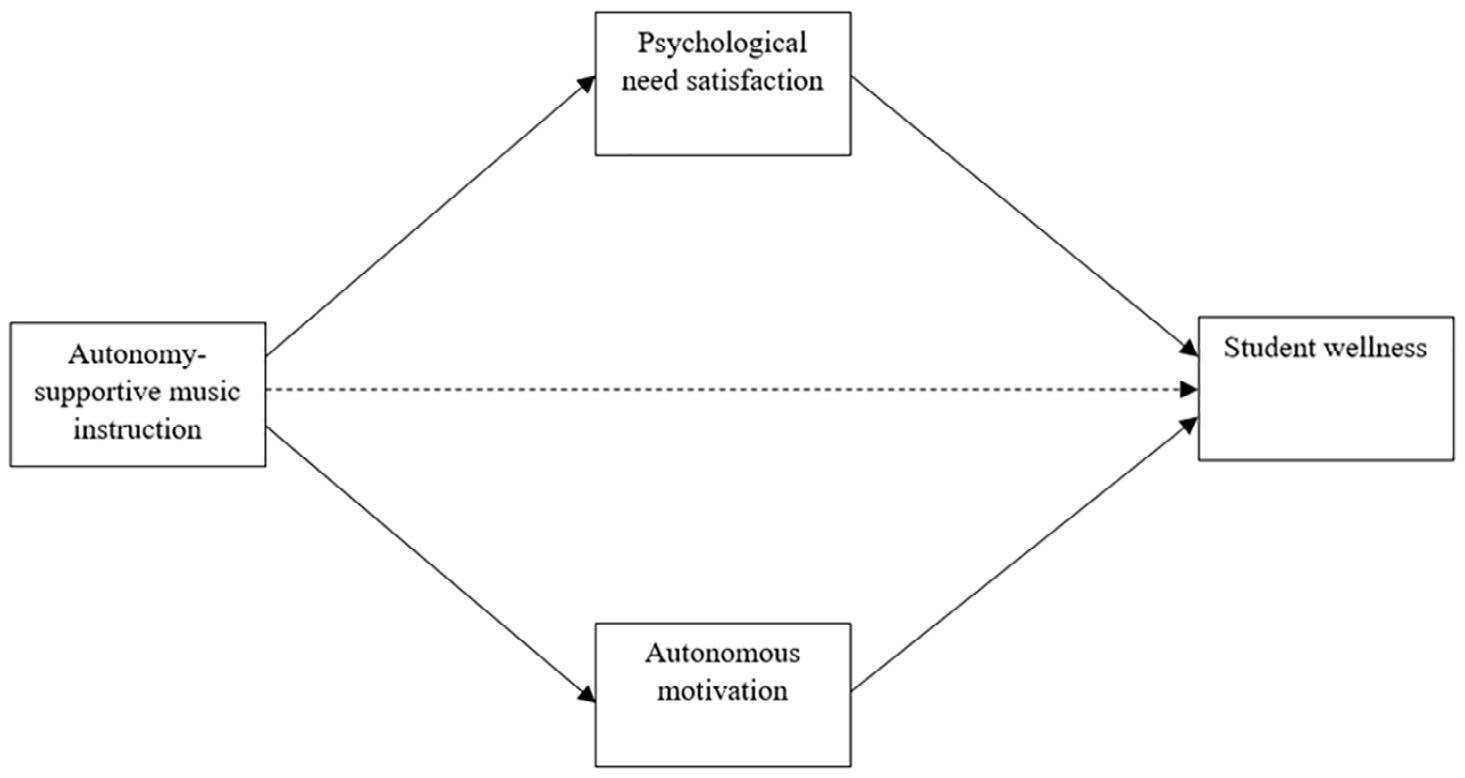
Proposed model.

## 4 Method

### 4.1 Sample and Sampling Design

The study was conducted in Chinese Mainland using a cluster‐randomized experimental design. Participants were undergraduate students enrolled in music‐related courses at public universities and specialized arts institutions. A multi‐stage sampling approach was employed (Etikan and Bala 2017). In the first stage, universities offering formal music programs were approached and institutional or departmental approval was obtained. In the second stage, intact music classes within the participating institutions were selected as the sampling units. This procedure enabled recruitment at both the institutional and classroom levels while preserving the naturally occurring instructional structure of university music education. To prevent instructional contamination, randomization was conducted at the class level (Li et al. 2024; Rutterford et al. 2015), with classes assigned to either the autonomy‐supportive music instruction condition or the control condition. All students within a class received the same instructional approach throughout the study period. Class‐level randomization was appropriate because the intervention was delivered through instructors to intact classes, whereas assigning individual students within the same class to different conditions could have resulted in treatment contamination (Li et al. 2024; Rutterford et al. 2015).

Data were collected using physical, paper‐based questionnaires administered during regular class sessions. Three waves of data collection were conducted with the same participants. At baseline, prior to the intervention, questionnaires were distributed to 720 students, of whom 688 returned usable responses. The second wave was administered during the intervention period, with 652 questionnaires returned. The final wave was conducted immediately after the intervention, yielding 628 responses. To enable matching across waves while preserving anonymity, participants generated a self‐identification code at the first wave and reproduced the same code at subsequent waves. After matching responses across all three waves and excluding cases with incomplete data, 612 students were retained for the final analysis, resulting in an overall usable response rate of approximately 85%. This longitudinal matching procedure allowed responses from the same participant to be linked across all three waves without collecting directly identifying information.

The final sample comprised 612 undergraduate students enrolled in music performance, music education, and music theory/composition programs. The demographic characteristics of the participants are presented in Table 1. Overall, the sample represented both genders, multiple academic years, varying levels of prior formal music training, and a broad age distribution, providing a diverse representation of university music students.

**TABLE 1:** Sample characteristics of participants.

Characteristic	Category	*n*	%
Gender	Male	288	47.1
	Female	324	52.9
Age (years)	18–19	159	26.0
	20–21	269	44.0
	≥ 22	184	30.0
Academic year	First	177	28.9
	Second	171	27.9
	Third	153	25.0
	Fourth or above	111	18.2
Music specialization	Music performance	214	35.0
	Music education	245	40.0
	Music theory/composition	153	25.0
Formal music training	< 5 years	214	35.0
	5–10 years	257	42.0
	> 10 years	141	23.0

*Note: N* = 612.

### 4.2 Intervention and Procedure

Prior to implementation, instructors assigned to the autonomy‐supportive music instruction (ASMI) condition participated in a structured 1‐day training workshop designed to familiarize them with the principles of autonomy‐supportive teaching derived from Self‐Determination Theory (Deci and Ryan 2000; Reeve and Cheon 2021). The workshop combined theoretical orientation with practical demonstrations and focused on implementing autonomy‐supportive teaching behaviors within university music education. Specifically, instructors were trained to provide meaningful choices where pedagogically appropriate, acknowledge students' perspectives, communicate meaningful rationales for learning activities, provide informational rather than controlling feedback, encourage student initiative, and minimize controlling instructional language (Butler 2022; Bonneville‐Roussy et al. 2020; Reeve and Cheon 2021). Written instructional guidelines and teaching examples were provided to facilitate consistent implementation throughout the intervention period.

The intervention was implemented over an 8‐week period during regularly scheduled music classes. Students in the autonomy‐supportive condition received the same curricular content and learning objectives as those in the control condition; however, instructors consistently incorporated autonomy‐supportive teaching strategies into their classroom instruction. These strategies included encouraging students to express their musical ideas, offering meaningful choices in selected learning activities where feasible, acknowledging students' perspectives during instruction, explaining the purpose of technically demanding exercises, and providing constructive, informational feedback that emphasized improvement rather than external evaluation (Bonneville‐Roussy and Evans 2025; Butler 2022; Hendriks et al. 2023; Xu and Li 2025).

Students assigned to the control condition continued to receive conventional music instruction delivered according to the existing curriculum and institutional teaching practices. The instructional content, teaching hours, learning objectives, and assessment requirements remained comparable across both conditions, ensuring that the primary distinction between groups was the instructional style rather than differences in curriculum or instructional time (Cheon et al. 2023; Matos et al. 2025).

To promote implementation consistency, instructors assigned to the intervention condition maintained regular communication with the research team throughout the 8‐week intervention. Fidelity of implementation was monitored through periodic classroom observations and instructor implementation checklists completed at regular intervals. These procedures were intended to ensure that the autonomy‐supportive teaching principles were implemented consistently across intervention classes while minimizing deviations from the planned instructional protocol (Reeve and Cheon 2021).

As shown in Table 2, baseline measures were collected before implementation of the intervention, motivational variables were assessed during the intervention period, and the primary outcome variable (student wellness) was measured immediately after completion of the intervention. This temporal separation strengthened the proposed causal ordering among the intervention, mediating mechanisms, and outcome variables.

**TABLE 2:** Study design and data collection procedure.

Study phase	Timing	Primary purpose	Variables collected
Preparation	One week before intervention	Instructor training, classroom randomization, intervention briefing	—
Wave 1	Week 0 (pre‐intervention)	Baseline assessment	Demographics; Student wellness
Intervention	Weeks 1–8	Autonomy‐supportive music instruction (experimental) or conventional music instruction (control)	—
Wave 2	Week 4	Mid‐intervention assessment	Psychological need satisfaction; Autonomous motivation
Wave 3	Week 8	Post‐intervention assessment	Student wellness; Manipulation check
Final dataset	After matching	Final multilevel analyses	*N* = 612

### 4.3 Measures

All study variables were assessed using established self‐report instruments adapted to the music education context. Items were rated on a five‐point Likert scale ranging from 1 (*strongly disagree*) to 5 (*strongly agree*). Higher scores indicated higher levels of the respective constructs. Scale adaptations involved minor wording changes to reflect music instruction while retaining the original meaning of the items. The wording of each adapted item was reviewed to ensure that it remained conceptually consistent with the original scale while being understandable within university music‐learning contexts.

Because the study was conducted in Chinese Mainland, all measurement instruments were translated into Chinese following Brislin's (1980) translation and back‐translation procedure. First, the original English scales were translated into Chinese by a bilingual researcher with expertise in educational psychology. A second independent bilingual translator then back‐translated the Chinese version into English without access to the original instruments. The original and back‐translated versions were subsequently compared, and any discrepancies were resolved through discussion among the translators and the research team to ensure semantic and conceptual equivalence. Finally, the translated questionnaire was pilot tested with a small group of university music students to confirm item clarity, readability, and contextual appropriateness before the main data collection.

#### 4.3.1 Autonomy‐Supportive Music Instruction

Autonomy‐supportive music instruction was operationalized as an experimental condition at the class level and therefore was not measured using a self‐report scale. To verify the effectiveness of the instructional manipulation, perceived autonomy support was assessed as a manipulation check. This construct was measured using a 6‐item version of the Learning Climate Questionnaire developed by Williams and Deci (1996). The scale assessed students' perceptions of their instructor's autonomy‐supportive behaviors, such as offering choice, acknowledging student perspectives, and using non‐controlling language. A sample item was “My instructor provides me with choices and options in how I learn music.” This measure was administered at the post‐intervention wave only and was used solely to confirm that the intervention produced meaningful differences in perceived teaching style between conditions. It was not treated as a mediator or substantive outcome in the hypothesized model. Because the manipulation check was intended to assess whether students experienced the instructional conditions differently after exposure to the intervention, it was administered only at the post‐intervention assessment.

#### 4.3.2 Psychological Need Satisfaction

Psychological need satisfaction was measured using 9 items adapted from the Basic Psychological Need Satisfaction scale proposed by Deci and Ryan (2000). The scale captured satisfaction of autonomy, competence, and relatedness needs within the music learning context, with three items representing each need. Sample items included “I feel free to express my musical ideas in this class,” “I feel capable of improving my musical skills,” and “I feel connected to others in this class.” Item responses were averaged to form a composite indicator of psychological need satisfaction. Although autonomy, competence, and relatedness represent conceptually distinguishable needs, the present study used an overall composite because its theoretical purpose was to assess the general need‐based process through which the instructional environment influenced student wellness. SDT research commonly conceptualizes the collective satisfaction of these needs as an indicator of whether the social environment supports optimal psychological functioning (Deci and Ryan 2000; Martela et al. 2023; Schutte and Malouff 2021). The composite therefore reflected students' overall experience of volition, effectiveness, and interpersonal connection within the music‐learning environment.

#### 4.3.3 Autonomous Motivation

Autonomous motivation was assessed using 6 items adapted from the Academic Motivation Scale developed by Vallerand et al. (1989), focusing on intrinsic motivation and identified regulation. These items captured students' self‐determined reasons for engaging in music learning activities. Sample items included “I learn music because I enjoy it” and “I study music because it is personally important to me.” Items were averaged to compute an overall autonomous motivation score. Combining intrinsic motivation and identified regulation was consistent with the SDT conceptualization of autonomous motivation because both forms of regulation involve engagement that is experienced as self‐endorsed, although they differ in whether the activity is undertaken for inherent enjoyment or personally accepted value (Ryan and Deci 2017).

#### 4.3.4 Student Wellness

Student wellness was measured as the primary outcome variable using 8 items adapted from the Warwick–Edinburgh Mental Well‐Being Scale developed by Tennant et al. (2007). The scale assessed students' positive psychological functioning and perceived mental well‐being in the academic context. A sample item was “I feel mentally well while studying at university.” Student wellness was measured at both baseline and post‐intervention, with baseline scores included as a control variable in subsequent analyses. In the present study, wellness referred to positive psychological functioning rather than the absence of psychological disorder. This conceptualization encompasses positive affective and functional experiences, including feeling mentally well, functioning effectively, and maintaining positive engagement with life, consistent with the orientation of the Warwick–Edinburgh scale (Tennant et al. 2007). Accordingly, the term wellness was used throughout the manuscript to denote positive psychological functioning and was distinguished from narrower indicators of distress, psychopathology, or temporary affective states.

#### 4.3.5 Control Variables

Demographic information, including gender, age, academic year, and prior music training experience, was collected at baseline and treated as control variables. Baseline student wellness was also included as a covariate in the models predicting post‐intervention wellness, allowing the estimated intervention effect to reflect differences in the outcome after accounting for participants' initial wellness levels.

All scales demonstrated acceptable internal consistency reliability in the present study, supporting their suitability for multilevel mediation analysis. The reliability coefficients for each construct are reported alongside the descriptive statistics and measurement results.

### 4.4 Data Analysis

Data analysis was conducted using IBM SPSS 29.0 for preliminary analyses and Mplus 8.9 for multilevel structural equation modeling (MSEM). Because students were nested within intact music classes that received the instructional intervention, multilevel analyses were employed to account for the hierarchical structure of the data and to obtain unbiased parameter estimates (Hox et al. 2018; Preacher et al. 2010). Prior to hypothesis testing, descriptive statistics, Pearson correlation coefficients, and internal consistency reliability coefficients (Cronbach's *α*) were computed for all study variables. Composite reliability (CR) and average variance extracted (AVE) were also calculated to evaluate construct reliability and convergent validity (Fornell and Larcker 1981; Hair et al. 2022).

To justify the use of multilevel modeling, unconditional null models were first estimated and intraclass correlation coefficients (ICC1 and ICC2) were calculated for the study variables. The ICC values indicated that a meaningful proportion of the variance occurred at the classroom level, supporting the use of multilevel analyses and the aggregation of the instructional intervention at the class level (Bliese 2000; Hox et al. 2018).

The effectiveness of the instructional manipulation was examined by comparing perceived autonomy support between the experimental and control conditions using the manipulation check measure. Independent‐samples *t*‐tests were conducted to verify that students in the autonomy‐supportive music instruction condition perceived significantly higher levels of autonomy support than those in the control condition.

The hypothesized relationships were examined using multilevel structural equation modeling with maximum likelihood estimation. Baseline student wellness together with gender, age, academic year, and prior music training were included as control variables in all structural models. Hypothesis 1 was examined by estimating the direct effect of autonomy‐supportive music instruction on post‐intervention student wellness. Hypotheses 2 and 3 were tested by estimating the effects of the intervention on psychological need satisfaction and autonomous motivation, respectively. Hypotheses 4 and 5 were evaluated through multilevel mediation analyses in which psychological need satisfaction and autonomous motivation were specified as parallel mediators linking autonomy‐supportive music instruction to student wellness.

The significance of the indirect effects was assessed using bias‐corrected bootstrap confidence intervals based on 5000 bootstrap resamples. Indirect effects were considered statistically significant when the 95% confidence interval did not include zero (Hayes 2022; Preacher et al. 2010). Model fit was evaluated using multiple fit indices, including the comparative fit index (CFI), Tucker–Lewis index (TLI), root mean square error of approximation (RMSEA), and standardized root mean square residual (SRMR). Following commonly accepted guidelines, CFI and TLI values of 0.90 or higher and RMSEA and SRMR values below 0.08 indicated acceptable model fit (Hu and Bentler 1999).

## 5 Results

All analyses accounted for the nested structure of the data, with students nested within classes. Accordingly, multilevel modeling was applied throughout.

### 5.1 Common Method Bias

Common method variance was examined using Harman's single‐factor test. An unrotated exploratory factor analysis showed that the first factor accounted for 31.8% of the total variance, which is below the commonly recommended threshold of 50% (Podsakoff et al. 2003). These results suggest that common method bias was unlikely to pose a serious threat to the validity of the findings.

### 5.2 Randomization and Manipulation Checks

Randomization and manipulation check results are reported in Table 3. Randomization checks indicated no significant differences between the ASMI and control conditions in baseline student wellness (*M*_control = 3.54, SD = 0.71; *M*_ASMI = 3.56, SD = 0.69), gender, age, academic year, or prior music training experience (all ns). These findings confirm that the cluster randomization procedure produced comparable groups prior to the intervention.

**TABLE 3:** Randomization and manipulation check results by experimental condition (*N* = 612).

Variable	Control (*n* = 306) *M*(SD)/%	ASMI (*n* = 306) *M*(SD)/%	Test result
Student wellness (baseline; T0)	3.54 (0.71)	3.56 (0.69)	ns
Gender (female %)	52.6%	53.4%	ns
Age (years)	20.9 (1.4)	21.0 (1.5)	ns
Academic year	2.32 (1.08)	2.29 (1.05)	ns
Prior music training (years)	7.4 (3.2)	7.6 (3.1)	ns
Perceived autonomy support	3.28 (0.62)	3.89 (0.58)	***

*Note:* Values are means with standard deviations in parentheses unless otherwise indicated.

Abbreviations: ASMI = autonomy‐supportive music instruction, ns = non‐significant.

*** *p* < 0.001.

The effectiveness of the instructional manipulation was assessed through students' perceived autonomy support. As shown in Table 2, students in the ASMI condition reported significantly higher perceived autonomy support (*M* = 3.89, SD = 0.58) than those in the control condition (*M* = 3.28, SD = 0.62), *p* < 0.001. This result confirms that the autonomy‐supportive instructional practices were successfully implemented.

### 5.3 Descriptive Statistics and Correlations

Descriptive statistics, group means, and bivariate correlations among the study variables are presented in Table 4. Inspection of the means indicated that students in the autonomy‐supportive music instruction (ASMI) condition reported higher levels of psychological need satisfaction, autonomous motivation, and post‐intervention student wellness than students in the control condition, whereas baseline student wellness was comparable across conditions. Standard deviations were moderate across all variables, indicating sufficient variability and no apparent ceiling or floor effects. Cronbach's alpha (*α*), composite reliability (CR), and average variance extracted (AVE) values all exceeded the commonly recommended thresholds, indicating satisfactory internal consistency reliability and convergent validity of the study measures (Fornell and Larcker 1981; Hair et al. 2022).

**TABLE 4:** Descriptive statistics, reliability estimates, group means, and bivariate correlations.

Variable	Control (*n* = 306) *M* (SD)	ASMI (*n* = 306) *M* (SD)	Total *M*	SD	*α*	CR	AVE	1	2	3	4
1. Psychological need satisfaction	3.58 (0.61)	3.86 (0.62)	3.72	0.63	0.89	0.92	0.64	—			
2. Autonomous motivation	3.57 (0.66)	3.79 (0.64)	3.68	0.66	0.87	0.91	0.62	0.56**	—		
4. Student wellness (T0)	3.54 (0.71)	3.56 (0.69)	3.55	0.70	0.91	0.93	0.66	0.32**	0.28**	—	
4. Student wellness (T2)	3.63 (0.69)	3.77 (0.66)	3.70	0.68	0.92	0.94	0.68	0.44**	0.39**	0.62**	—

Abbreviations: *α* = Cronbach's alpha, ASMI = autonomy‐supportive music instruction (0 = control, 1 = intervention), AVE = average variance extracted, CR = composite reliability.

** *p* < 0.01.

Correlation analyses showed that psychological need satisfaction was positively associated with autonomous motivation (*r* = 0.56, *p* < 0.01) and post‐intervention student wellness (*r* = 0.44, *p* < 0.01). Autonomous motivation was also positively associated with post‐intervention student wellness (*r* = 0.39, *p* < 0.01). Baseline student wellness was strongly associated with post‐intervention student wellness (*r* = 0.62, *p* < 0.01), indicating substantial temporal stability. No correlation exceeded 0.70, suggesting that multicollinearity was unlikely to be a concern. The correlations reported in Table 4 are individual‐level, zero‐order correlations provided for descriptive purposes; all hypothesis tests were conducted using multilevel analyses that accounted for students being nested within classes.

### 5.4 Intraclass Correlation Coefficients and Multilevel Justification

Prior to hypothesis testing, unconditional null models were estimated to evaluate the extent of between‐class variation in the study variables. As shown in Table 5, the ICC(1) values ranged from 0.09 to 0.12, indicating that a meaningful proportion of the variance was attributable to differences between classes. Furthermore, ICC(2) values ranged from 0.69 to 0.73, demonstrating acceptable reliability of class‐level means. Consistent with the design effects exceeding the recommended threshold of 1.50, these findings supported the use of multilevel analyses for hypothesis testing (Bliese 2000; Hox et al. 2018).

**TABLE 5:** Interclass correlation coefficients and multilevel justification.

Variable	ICC(1)	ICC(2)	Design effect	Interpretation
Psychological need satisfaction	0.12	0.73	2.10	Adequate between‐class variance; multilevel analysis justified
Autonomous motivation	0.10	0.71	1.96	Adequate between‐class variance; multilevel analysis justified
Student wellness (T2)	0.09	0.69	1.84	Meaningful clustering; multilevel analysis appropriate

*Note:* ICC(1) = intraclass correlation coefficient, representing the proportion of variance attributable to differences between classes. ICC(2) = reliability of class‐level means. Design effect was calculated as 1 + (average cluster size − 1) × ICC(1).

### 5.5 Direct Effects of Autonomy‐Supportive Music Instruction

The direct effects of autonomy‐supportive music instruction on the proposed mediators and outcome variables were examined using multilevel regression analyses. Results are presented in Table 6. Autonomy‐supportive music instruction had a significant positive effect on psychological need satisfaction (*β* = 0.34, SE = 0.05, *p* = 0.001) and autonomous motivation (*β* = 0.27, SE = 0.06, *p* = 0.001), supporting Hypotheses 2 and 3.

**TABLE 6:** Multilevel path estimates for direct and mediator effects predicting student wellness (*N* = 612).

Dependent variable	Predictor	Estimate (*β*)	SE	*p*
Psychological need satisfaction	Autonomy‐supportive music instruction	0.34	0.05	< 0.001
Autonomous motivation	Autonomy‐supportive music instruction	0.27	0.06	< 0.001
Student wellness (post; T2)	Autonomy‐supportive music instruction	0.18	0.07	0.010
Student wellness (post; T2)	Psychological need satisfaction	0.24	0.05	< 0.001
Student wellness (post; T2)	Autonomous motivation	0.19	0.05	< 0.001
Student wellness (post; T2)	Baseline student wellness (T0)	0.56	0.04	< 0.001

*Note:* Estimates are standardized coefficients from multilevel regression analyses with students nested within classes. Autonomy‐supportive music instruction was coded 0 = control and 1 = intervention.

Autonomy‐supportive music instruction also exerted a significant total effect on post‐intervention student wellness (*β* = 0.18, SE = 0.07, *p* = 0.010) when controlling for baseline wellness. Baseline student wellness remained a strong predictor of post‐intervention wellness (*β* = 0.56, SE = 0.04, *p* < 0.001). These findings provide support for Hypothesis 1 and establish the prerequisite conditions for mediation testing.

### 5.6 Mediation Analyses

Multilevel mediation analyses were conducted to examine whether psychological need satisfaction and autonomous motivation mediated the relationship between autonomy‐supportive music instruction and student wellness. Bootstrapped indirect effects with 5000 resamples are reported in Table 7. Psychological need satisfaction significantly mediated the relationship between autonomy‐supportive music instruction and student wellness (indirect effect *β* = 0.082, Boot SE = 0.019, 95% CI [0.047, 0.122], *p* < 0.000). Autonomous motivation also served as a significant mediator (indirect effect *β* = 0.051, Boot SE = 0.016, 95% CI [0.023, 0.085], *p* = 0.001).

**TABLE 7:** Multilevel indirect effects of autonomy‐supportive music instruction on student wellness through psychological need satisfaction and autonomous motivation (*N* = 612).

Effect	Indirect/direct estimate (*β*)	Boot SE	95% CI (LL, UL)	*p*
Indirect effect via psychological need satisfaction	0.082	0.019	(0.047, 0.122)	< 0.001
Indirect effect via autonomous motivation	0.051	0.016	(0.023, 0.085)	0.001
Total indirect effect	0.133	0.024	(0.089, 0.182)	< 0.001
Direct effect (c′)	0.047	0.058	(−0.067, 0.160)	0.418
Total effect (c)	0.180	0.070	(0.040, 0.319)	0.010

*Note:* Indirect effects and confidence intervals were estimated using bootstrapping (5000 resamples) within a multilevel framework with students nested within classes.

The total indirect effect was statistically significant (*β* = 0.133, Boot SE = 0.024, 95% CI [0.089, 0.182], *p* < 0.001). When both mediators were included in the model, the direct effect of autonomy‐supportive music instruction on student wellness was no longer significant (*β* = 0.047, SE = 0.058, *p* = 0.418), whereas the total effect remained significant (*β* = 0.180, SE = 0.070, *p* = 0.010). According to established mediation criteria, this pattern indicates full mediation (Figure 2), whereby the effect of autonomy‐supportive music instruction on student wellness operates entirely through psychological need satisfaction and autonomous motivation.

**FIGURE 2:**
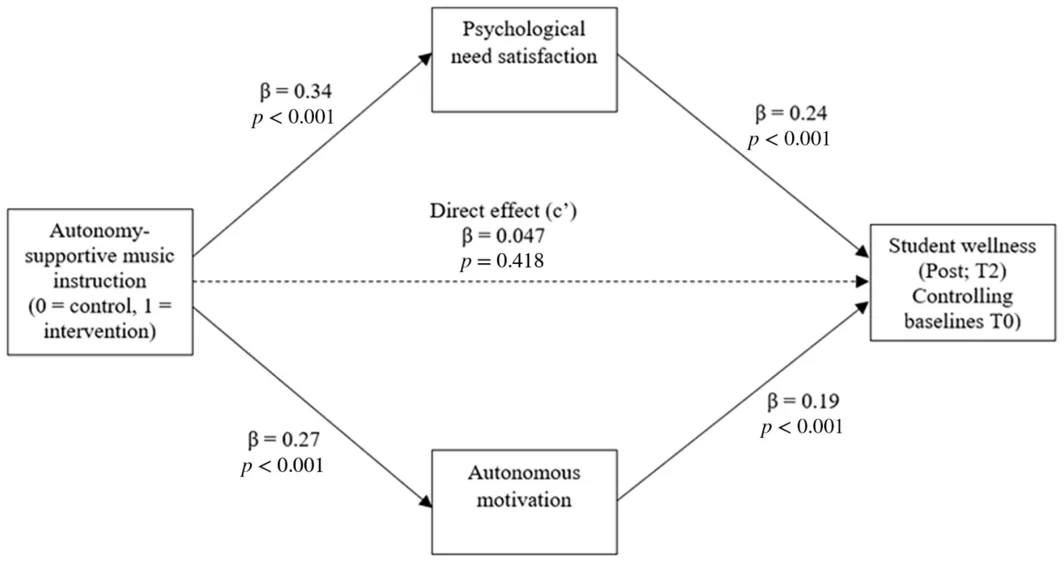
Multilevel SEM path diagram.

## 6 Discussion and Implications

The primary objective of this study was to examine whether autonomy‐supportive music instruction can enhance student wellness and to identify the psychological mechanisms underlying this relationship. Specifically, drawing on self‐determination theory, the study tested whether psychological need satisfaction and autonomous motivation mediate the effect of autonomy‐supportive instruction on student wellness using a cluster‐randomized experimental design. By integrating longitudinal measurement, multilevel modeling, and mediation analysis, the study aimed to provide more rigorous evidence for instructional processes that support student well‐being in higher education music contexts. Unlike the predominantly cross‐sectional evidence available in this literature, the present design strengthens confidence in the temporal ordering of the proposed motivational mechanisms while accounting for the nested structure of students within music classes (Li et al. 2024; Rutterford et al. 2015; Xu and Li 2025).

The findings demonstrate that autonomy‐supportive music instruction significantly enhanced students' psychological need satisfaction and autonomous motivation, which in turn predicted higher levels of student wellness. These results are consistent with self‐determination theory's central proposition that social contexts supporting autonomy, competence, and relatedness foster internalized motivation and psychological well‐being (Deci and Ryan 2000). Prior research has repeatedly shown that autonomy‐supportive teaching is positively associated with students' need satisfaction and autonomous motivation across educational domains (Guoxia and Yang 2024; Muth and Lüftenegger 2024; Reeve and Cheon 2024). The present study extends this literature by demonstrating these effects experimentally within university‐level music education, a context characterized by high performance demands and emotional investment. The findings therefore suggest that instructional style is not merely a background feature of music education but a consequential educational resource that can shape students' motivational experiences and psychological functioning (Bonneville‐Roussy et al. 2020; Butler 2022; Reeve and Cheon 2021).

Importantly, the mediation results revealed that the relationship between autonomy‐supportive instruction and student wellness was consistent with indirect‐only effects through psychological need satisfaction and autonomous motivation. This finding aligns with previous work suggesting that autonomy support does not directly produce well‐being outcomes but operates through motivational processes (Li et al. 2025; Zhang et al. 2022). While some correlational studies have reported direct associations between autonomy support and well‐being (Fülöp et al. 2025; Solms et al. 2025), the current experimental evidence suggests that such direct effects may diminish once core motivational mechanisms are accounted for. This helps reconcile inconsistencies in the literature and underscores the importance of testing mediation rather than relying solely on direct effects. From an SDT perspective, this pattern reinforces the proposition that supportive instructional environments contribute to wellness because they alter students' need experiences and reasons for engagement rather than exerting an independent psychological influence (Deci and Ryan 2000; Ryan and Deci 2017).

The positive association between psychological need satisfaction and student wellness observed in this study corroborates a large body of empirical research linking need fulfillment to mental health, vitality, and life satisfaction in educational settings (Butler 2022; Hernández et al. 2022). In music education specifically, where students often experience performance anxiety and evaluative pressure, satisfying needs for autonomy and competence appears particularly critical for sustaining psychological well‐being. The present findings therefore extend prior research by demonstrating that need satisfaction serves not only as a predictor of engagement or achievement, but also as a central mechanism for promoting student wellness in performance‐oriented learning environments. This finding further suggests that efforts to improve student wellness should extend beyond reducing distress and should also create learning environments that consistently support autonomy, competence, and relatedness (Hatfield et al. 2025; Martela et al. 2023; Schutte and Malouff 2021).

Similarly, autonomous motivation emerged as a significant mediator between instructional style and student wellness. This finding aligns with studies showing that intrinsically motivated or personally valued learning is associated with greater emotional well‐being and lower stress (Lo 2024). By contrast, controlled forms of motivation have been linked to exhaustion and maladaptive outcomes (Lee et al. 2023; Zhang et al. 2025). The current results suggest that autonomy‐supportive music instruction facilitates wellness by encouraging students to engage in music learning for self‐endorsed reasons, rather than external pressure or obligation. For music students, whose learning often requires prolonged practice and repeated performance evaluation, engaging for personally endorsed reasons may enable demanding learning activities to be experienced as meaningful challenges rather than externally imposed burdens (Bonneville‐Roussy and Evans 2025; Koehler et al. 2023; Ryan and Deci 2017).

Beyond aligning with prior studies, the present research makes several novel contributions. First, it extends autonomy‐supportive teaching research into music education using an experimental design, addressing a notable gap in the literature, which has relied largely on cross‐sectional or qualitative approaches in this domain (Gülbak and Gülbak 2025; Wijaya 2023). Second, by positioning student wellness as the primary outcome, the study broadens the scope of instructional effectiveness beyond performance and achievement, responding to growing concerns about student mental health in higher education (Basri 2023). Third, the use of a cluster‐randomized design and multilevel mediation analysis strengthens causal inference and enhances methodological rigor relative to prior work. Finally, by examining psychological need satisfaction and autonomous motivation simultaneously, the study provides a more comprehensive explanation of the distinct motivational processes through which autonomy‐supportive instruction contributes to student wellness (Deci and Ryan 2000; Ryan and Deci 2017).

The findings also contribute to cross‐cultural motivation research. Although autonomy‐supportive practices are sometimes assumed to be less effective in collectivist or hierarchical cultures, growing evidence suggests that basic psychological needs are universal, even if their expression varies culturally (Deniz et al. 2025). The present study supports this perspective by demonstrating that autonomy‐supportive instruction benefits Chinese university students' motivational functioning and wellness, challenging simplistic cultural dichotomies and reinforcing the cross‐cultural applicability of self‐determination theory. These findings further suggest that autonomy‐supportive pedagogy can operate within structured and hierarchical educational environments without requiring the removal of instructional guidance or academic expectations (Lam 2023; Reeve and Cheon 2021).

In summary, this study advances theory and research by demonstrating that autonomy‐supportive music instruction promotes student wellness through psychological need satisfaction and autonomous motivation. By clarifying these mechanisms using experimental evidence in a non‐Western context, the study deepens understanding of how instructional practices can support both learning and well‐being in higher education.

The findings of this study suggest that autonomy‐supportive instructional practices can serve as an effective and low‐cost strategy for promoting student wellness in university music education. Music instructors can enhance students' psychological well‐being by deliberately incorporating autonomy‐supportive behaviors into daily teaching practices, such as offering meaningful choices in repertoire selection, acknowledging students' perspectives during feedback, and providing rationales for technically demanding exercises. These practices can be incorporated into routine instruction without substantial curricular restructuring because autonomy support concerns how instructors communicate, provide feedback, and involve students in learning rather than whether formal standards are removed.

The results further highlight the importance of targeting students' psychological need satisfaction as a proximal instructional goal. Teacher training programs in music education may benefit from explicitly addressing how instructional behaviors support students' autonomy, competence, and relatedness, as need satisfaction was shown to be a central mechanism linking teaching style to wellness outcomes. Professional development programs may therefore include practical training in perspective acknowledgment, meaningful choice provision, informational feedback, rationale provision, and relationship‐building practices that support all three psychological needs.

In addition, the mediating role of autonomous motivation suggests that music programs should move beyond performance‐driven evaluation systems that rely heavily on external pressure and comparison. Designing learning environments that emphasize personal growth, intrinsic enjoyment, and meaningful engagement with music may reduce stress and enhance students' psychological resilience. Balancing performance expectations with opportunities for self‐directed learning and reflective practice may help students sustain long‐term motivation while reducing unnecessary pressure associated with externally controlled engagement.

Last but not least, at an institutional level, the findings provide empirical support for integrating autonomy‐supportive pedagogy into broader student well‐being and mental health initiatives. Rather than treating wellness as separate from academic instruction, universities may consider instructional reform as part of a preventative approach to student mental health, particularly in high‐pressure disciplines such as music education. Such initiatives may be especially valuable in performance‐oriented programs because instructors' day‐to‐day practices can influence not only students' learning outcomes but also their motivational experiences and psychological functioning.

Several limitations of this study should be acknowledged. First, although the cluster‐randomized experimental design strengthens causal inference, the intervention was implemented over a relatively short instructional period. Student wellness and motivational processes may evolve over longer time spans, particularly in music education where learning and identity development are gradual. Future research should examine the long‐term sustainability of autonomy‐supportive instructional effects using extended or repeated interventions. In particular, longitudinal follow‐up assessments conducted several months after the intervention would help determine whether improvements in psychological need satisfaction, autonomous motivation, and wellness persist beyond the immediate post‐intervention period.

Second, the study relied primarily on student self‐report measures. While the multi‐wave design reduces concerns related to common method bias, future studies could incorporate additional data sources, such as classroom observations or behavioral indicators, to provide a more comprehensive assessment of instructional processes and wellness outcomes. The use of student reports for both the mediators and wellness may still inflate associations through shared method variance, despite the temporal separation of measurements. Future studies could therefore combine student surveys with independent classroom observations, instructor reports, practice records, performance indicators, or physiological markers of stress (Podsakoff et al. 2003; Tennant et al. 2007).

Third, perceived autonomy support was assessed only after the intervention. Although this measure served its intended purpose as a manipulation check, the absence of a pre‐intervention assessment prevents direct examination of within‐student change in perceived autonomy support. Future research should measure perceived instructional support both before and after intervention implementation to provide a stronger test of treatment‐induced change.

Fourth, the study operationalized psychological need satisfaction as a composite of autonomy, competence, and relatedness. This approach was consistent with the study's focus on the general need‐based mechanism proposed by SDT, but it may conceal differential effects across the three needs (Deci and Ryan 2000; Martela et al. 2023). Future studies should test the needs separately to determine whether autonomy‐supportive music instruction has stronger effects on autonomy than on competence or relatedness, particularly in highly evaluative music‐learning environments.

Fifth, the theoretical model focused on autonomous motivation and need satisfaction but did not include controlled motivation, need frustration, competence‐supportive and relatedness‐supportive teaching, or controlling instructional behavior. These omitted constructs may provide a more complete account of how music instruction influences wellness. Future research should examine whether autonomy‐supportive practices operate differently from controlling practices and whether need frustration and controlled motivation explain maladaptive outcomes such as anxiety, exhaustion, or disengagement.

Sixth, although the intervention was designed to isolate instructional style, other unmeasured classroom factors may have contributed to the observed effects. Teacher enthusiasm, interpersonal warmth, peer climate, classroom cohesion, and differences in performance expectations could have influenced students' motivational experiences and wellness. Future studies should measure these factors explicitly and test whether the effects of autonomy‐supportive instruction remain robust after accounting for them.

Finally, the sample was drawn from university music programs in China, which may limit the generalizability of the findings. Future research could replicate the model across different cultural contexts, educational levels, and academic disciplines to further test the robustness of the observed relationships. Replication in conservatories, secondary‐school music programs, private music academies, and Western educational settings would clarify whether the observed effects generalize across institutional and cultural contexts. Such work could also examine whether the meaning and implementation of autonomy support vary under different norms of teacher authority, hierarchy, and artistic training (Lam 2023; Xu and Li 2025).

## 7 Conclusion

This study provides experimental evidence that autonomy‐supportive music instruction enhances student wellness by fostering psychological need satisfaction and autonomous motivation. Using a cluster‐randomized design and multilevel mediation analysis in university music classrooms, the findings demonstrate that instructional practices emphasizing choice, rationale, and student perspective‐taking promote well‐being through motivational mechanisms rather than through direct effects alone. Specifically, the findings indicate that the beneficial influence of autonomy‐supportive instruction on student wellness is transmitted through students' satisfaction of their basic psychological needs and the development of more autonomous forms of motivation, thereby providing empirical support for the motivational processes proposed by Self‐Determination Theory.

By extending autonomy‐supportive teaching research to music education and positioning student wellness as a central outcome, the study highlights the dual role of instructional practices in supporting both learning and psychological health. Furthermore, by employing a cluster‐randomized experimental design within a non‐Western higher education context, the study strengthens the methodological and cross‐cultural evidence supporting the application of autonomy‐supportive pedagogy in music education.

Overall, the findings underline the importance of integrating autonomy‐supportive pedagogy into higher education as a viable approach to promoting student wellness in demanding academic contexts. Beyond improving educational outcomes, autonomy‐supportive teaching may contribute to healthier and more sustainable learning environments by fostering students' motivational resources and psychological well‐being. The present findings therefore encourage educators and institutions to recognize instructional practice as an important component of student wellness initiatives, particularly within performance‐oriented disciplines such as music education.

## Funding

The author has nothing to report.

## Ethics Statement

This study is conducted in accordance with the ethical principles of the Declaration of Helsinki. Ethical approval is obtained from the Institutional Review Board (IRB)/Research Ethics Committee of College of Arts and Design, Zhejiang A&F University prior to data collection. All study procedures are reviewed and approved to ensure the protection of participants' rights, privacy, and well‐being.

## Consent

All respondents provided informed consent before participating in the study. Participation was voluntary, and all responses were collected anonymously and treated confidentially for research purposes only.

## Conflicts of Interest

The author declares no conflicts of interest.

## Data Availability

The data and materials used in this article are confidential and can be requested by the author.
